# RBT-GA: a novel metaheuristic for solving the multiple sequence alignment problem

**DOI:** 10.1186/1471-2164-10-S1-S10

**Published:** 2009-07-07

**Authors:** Javid Taheri, Albert Y Zomaya

**Affiliations:** 1 School of Information Technologies, J12, The University of Sydney, Sydney, NSW 2006, Australia

## Abstract

**Background:**

Multiple Sequence Alignment (MSA) has always been an active area of research in Bioinformatics. MSA is mainly focused on discovering biologically meaningful relationships among different sequences or proteins in order to investigate the underlying main characteristics/functions. This information is also used to generate phylogenetic trees.

**Results:**

This paper presents a novel approach, namely RBT-GA, to solve the MSA problem using a hybrid solution methodology combining the Rubber Band Technique (RBT) and the Genetic Algorithm (GA) metaheuristic. RBT is inspired by the behavior of an elastic Rubber Band (RB) on a plate with several poles, which is analogues to locations in the input sequences that could potentially be biologically related. A GA attempts to mimic the evolutionary processes of life in order to locate optimal solutions in an often very complex landscape. RBT-GA is a population based optimization algorithm designed to find the optimal alignment for a set of input protein sequences. In this novel technique, each alignment answer is modeled as a chromosome consisting of several poles in the RBT framework. These poles resemble locations in the input sequences that are most likely to be correlated and/or biologically related. A GA-based optimization process improves these chromosomes gradually yielding a set of mostly optimal answers for the MSA problem.

**Conclusion:**

RBT-GA is tested with one of the well-known benchmarks suites (BALiBASE 2.0) in this area. The obtained results show that the superiority of the proposed technique even in the case of formidable sequences.

## Background

### Multiple Sequence Alignment

Biologists have always tried to understand the basic roles of nucleotides and genes [1]. One popular approach in trying to understand the function of a newly found gene or protein is to compare it with already known genes/sequences. A very common practice is to attempt to find one or more sequences in existing literature or databases that are reasonably close to the sequence in question. However, due to the fact that the number of known sequences is rapidly increasing every year, new classes of algorithms that are able to search massive data sets are needed.

Sequence Alignment algorithms are techniques that are used to find similarity among several DNA/Protein sequences. These algorithms are classified into two main categories: Pair-wise and MSA algorithms, each designed for a special purpose. In Pair-wise algorithms, the main goal is to find the similar or closely related parts (motifs) of two sequences; whereas in MSA, the main goal is to find the consensus parts of more than two sequences. Therefore, Pair-wise algorithms are mainly used to find similar sequences in a database; MSAs are mainly used to find the relationship among several sequences.

Several algorithms and techniques have already been suggested to solve this problem in each of the above two categories. Among these techniques, there exist several classical methods, like Dynamic Programming (DP), that can always find the optimal alignment for any two sequences (Pair-wise). However, these techniques cannot always be generalized to MSA cases (due to the excessive computation that is incurred after the addition of each extra sequence). Therefore, using classical methods in the MSA case is almost impossible. In fact, because it has been shown that MSA is NP-Complete [2], heuristics are mainly used to solve this problem.

Regardless of the solution methodology, MSAs can be categorized into three main solution categories: exact, progressive and iterative [3]. In exact methods, which are usually the generalized methods of the Needleman and Wunsch algorithm [4], all sequences are aligned simultaneously to find the optimal answer. The main drawback of this class of algorithms is their massive computational need, usually impossible to find the answer in polynomial time. In progressive algorithms, sequences are first aligned two-by-two (using an appropriate pair-wise algorithm) before finding the final alignment. Then, an alignment guidance tree is generated based on these pair-wise alignment scores. Sequences are combined step by step to find the optimal answer by starting from the closest two sequences. In this case, current sequences are modified to get the best fit for new combining sequences. Although this class of algorithms normally manages to find reasonable alignments (especially for generating phylogeny trees), their main disadvantage is their sensitivity for getting trapped into local minima. In iterative methods, all sequences are aligned simultaneously. By using one or more heuristic algorithms, an initial answer is computed first. Then, this initial answer is improved iteratively by using intelligent routines designed for this type of MSAs. Although these algorithms are not as sensitive as progressive algorithm to falling into local minima, however, they have their own drawbacks. For example, the accuracy of the final answer is greatly dependent on the quality of the seed solution.

### Related works

A number of alignment algorithms have been proposed to solve the MSA problem, such as MULTALIGN [5], MULTAL [6], PILEUP [7] and CLUSTALX [8], which provides a graphical interface for CLUSTALW [9]. They all use a global alignment algorithm in [4] to construct an alignment for the entire length of the sequences. The main difference among these methods is in the order they combine the input sequences. MULTAL deploys a sequential branching method to align the two closest sequences before building up the final alignment by subsequently aligning the next closest sequence to it. MULTALIGN and PILEUP construct a guide tree using UPGMA [10]. This tree is then used to align larger and larger groups of input sequences. CLUSTALX that uses the alternative neighbor-joining algorithm [11] to construct a guide tree has one of the most sophisticated scoring systems. It considers sequence weighting, position dependant gap penalties, and the automatic switching among scoring matrices based on the degree of similarity among the input sequences. PIMA [12] uses a local DP algorithm to align only the most conserved motifs. Two versions of this method have been developed, ML_PIMA and SB_PIMA, and they differ in the way they order the combination of input sequences and maximum linkage and sequential branching algorithms. DIALIGN [13] employs local alignment based on segment-to-segment comparison to construct the final alignment. Then, an iterative procedure is deployed to combine these segments toward generating the final alignment. PRRP [14] iteratively divides the input sequences into two groups and then subsequently realign them using a global group-to-group alignment algorithm. SAGA [15] evolves a population of alignments in a quasi evolutionary manner to gradually improve their fitness. MAFFT [16] identifies the homologous regions by a Fast Fourier Transform (FFT) approach. Using its simplified scoring matrix, MAFFT manages to significantly reduce the CPU time and increases the accuracy of alignments even for sequences having large insertions and extensions as well as distantly related sequences of similar length. ProbCons [17], which computes posterior-probability matrices and expected accuracies for each Pair-wise comparison, applies the probabilistic consistency transformation, and then computes an expected accuracy guide tree to progressively generate the final alignment. T-Coffee [18] pre-processes a data set of all pair-wise alignments between the input sequences to generate a guide tree for the progressive alignment. T-Coffee not only does focus on the next aligned sequences but also on the whole set of input sequences. MUSCLE [19] as one of the very fast algorithms in this field has three stages: draft progressive, improved progressive, and refinement. At the completion of each stage, a multiple alignment is available and the algorithm can be terminated. The first stage builds a progressive alignment, the second stage that might be iterated attempts to improve the tree and builds a new progressive alignment according to this tree, and, the third stage performs iterative refinement using a variant of tree-dependent restricted partitioning. MUMMALS [20] uses probabilistic consistency and improves its alignment quality by using Pair-wise alignment Hidden Markov models (HMMs). Parameters for such models have been estimated from a large library of structure-based alignments. There are also other HMMs methods that use statistical models of the primary structure consensus to align input sequences [21,22]. HMMT [23] uses the simulated annealing algorithm to maximize the probability that an HMM represents the sequences to be aligned. RBT [24,25] is another iterative algorithm that uses the *n*-dimensional version of the DP table (*n *is the number of input sequences) to find the best alignment among input sequences. The analogy of a Rubber Band is a unique contribution of this work.

Further, GA based algorithms were among the some of the most effective approaches used to solve the MSA problem. In [26], a combination of a GA and DP is used with two different distance matrices. The main drawback of this technique is its limitation in performing crossover and mutation operations. In [27], a GA approach is proposed with a description of the so-called Center Star Algorithm (CSA). In addition to this algorithm's convergence problems, forcing the GA to work around the CSA and the initial population creates a major disadvantage for this approach. It leads to the inability of the main search algorithm to explore all parts of the solution space. In [28], a very different GA approach is presented. In this work, five mutation operators are designed to be randomly selected in each cycle of the algorithm. Here, no particular optimization plan is used; therefore, this greedy algorithm just moves toward any potential answer. One of the most appropriate GA approaches to solve the MSA problem is presented in [29]. Although, the authors carefully define their chromosome, crossover and mutation operators, the definition of their scoring function appears to be their main drawback. In [30] a very interesting divide-and-conquer GA based approaches is presented. Here, the sequences are divided into smaller sequences and then they are aligned by a GA. If these partial alignments generate better results, they would be replaced by the original ones. Although this approach managed to significantly reduce the simulation time, there is no guarantee that the aggregation of these partially optimal strings ends up with the global minimum and/or a reasonable alignment.

In [31], the authors present a very simple implementation of the GA. In this work, the GA's convergence speed is significantly reduced by the simplicity of the algorithm. The fact that this GA approach discards many offspring is the main reason for its slow convergence. In [32], the convergence speed of a GA is increased by combining it with a Simulated Annealing algorithm. The GA in [3] use quantum mechanics concepts by employing a binary matrix to represent only four chromosomes that are used to solve the problem. In each GA cycle, the best three solutions are directly copied to the next generation and only one of them (the worst one) is selected for the GA operations. The proposed GA is significantly biased toward good answers, which strongly prevents it from exploring other parts of the solution space. Authors of the research in [33] present a GA based approach to find the optimal cut-off-points to divide the large sequences to several smaller ones. Each of these smaller sequences is solved by an Ant-Colony approach. The limited use of the GA just to find the cut-off-points is quite time consuming in this approach.

In this paper, a novel approach, RBT-GA, is presented to solve the MSA problem. The rest of the paper is structured as follows. The next section describes the problem, fundamentals of GA and RBT, and shows how RBT and GA are combined to solve the MSA problem. This is followed by simulation results, discussion and analysis, and conclusion.

## Methods

### Problem statement

Let {*S*_1 _*S*_2 _... *S*_*N*_} be *N *sequences over the alphabet set ψ, which contains 4 and 20 characters of DNA and Proteins sequences, respectively. Also, let ψ' = ψ ∪ {–} be the superset of ψ with and extra character for a 'gap'. The MSA problem can be defined as finding  with the following properties:

1.  = *S*_*i *_for all *i *= 1, 2,⋯, *N *providing that all gaps are removed from .

2.  where || denotes the length of .

3. The alignment score,  is maximized where  denotes a quotation of similarity between  and , and, *g*() is related to gaps of .

Based on the above, the MSA can be formulated as an optimization problem. However, it is important to note that the complexity of the problem increases exponentially as we add more sequences to the input sequences set – finding the optimal answer is not always possible. Thus, this is why classical methods like DP and Needleman's algorithm can only deal with a small number of short sequences.

### Genetic Algorithms

#### A Genetic algorithm optimizer

**Figure 1:**
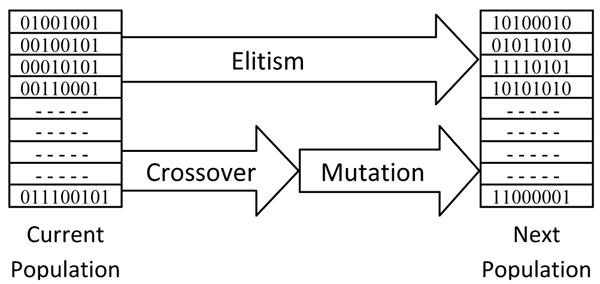
**Overall GA procedure**.

Metaheuristics are powerful classes of optimization techniques. A popular class among these techniques is GAs that are very robust search methods [34,35]. The overall procedure of a typical GA is given in Figure 1 though in some cases modifications might be needed when targeting certain problems, such as the case in this work.

#### Initial population

A GA is always initiated with a set of possible solutions of the problem, known as *initial population*. The initial population consists of several chromosomes. Each chromosome is formed from a series of binary or decimal numbers representing genes. The initial population is normally constructed by generating several random chromosomes that are supposed to represent the solution space rather homogenously. This attribute is much more important that the quality of the individual chromosomes in the initial population. During the optimization process, the chromosomes are evaluated by the genetic optimizer and the best of them are selected to generate the next population. In fact, obtaining the optimal answer relies on appropriately generating the future populations.

#### The fitness function

In all optimization problems, the aim is to minimize or maximize a given cost function. Similarly, in a GA, a function known as *fitness function *is used to evaluate the goodness of each chromosome. This function assigns a positive number to each chromosome (each possible solution) to represent the level of its goodness or badness. In maximization problems, the fitness function is usually proportional to the main cost function, while, in minimization problems, it is usually inversely proportional to it.

#### Selecting the best chromosomes

As mentioned earlier, the only way to evaluate chromosomes of a population is by their fitness values. In GA, the best chromosomes from the current population are selected to produce the next population. The chromosomes are selected so that those with higher fitness values have more chances of being selected. To achieve this, a *roulette wheel *from the current chromosomes is constructed such that the surface (number of slots) assigned to each chromosome is proportional to its fitness value. Then, the wheel is spun and the chromosome that the wheel picks is selected.

#### Recombining parent chromosomes

Several operators are used to generate the next population from the current one. In each optimization cycle, one or two parents are used to generate the offspring chromosomes. These combinations consist of three main operations: crossover, mutation, and elitism.

#### Crossover

This operator is used to exchange the genetic materials (genes) between two parents and create one or two offspring. Here, two chromosomes are broken at random positions, then, the genes between these breakpoints are exchanged with a given probability.

#### Mutation

In this operation, several genes of a chromosome are randomly selected and their values are mutated (changed).

#### Elitism

It is possible sometimes to lose the best solution that the algorithm has already found after performing crossover and mutation for several iterations. To prevent this, some of the best chromosomes from the current population are directly copied into the next generation without any modification, i.e. crossover and mutation. This ensures the existence of good chromosomes in each generation even if low quality chromosomes emerge for one reason or another.

### Terminating the algorithm

A criterion (or a combination of criteria) must be selected to terminate the optimization process. The common criteria used in the case of GAs are: total time, number of iterations, best chromosome fitness value, minimum improvement of the best chromosome, and minimum relative improvement of the best chromosome.

### Overall procedure of a GA

#### Rubber Band Technique

The Rubber Band Technique is an iterative heuristic to solve the MSA[24,25]. In this approach, which is inspired by the general behavior of a rubber band on a plate with poles, an initial answer is generated before launching the main optimization procedure. Using several operators, this initial answer is modified iteratively to obtain better alignment scores. The following definitions are essential for the clarification of this optimization procedure.

#### Grid Answer Space

**Figure 2:**
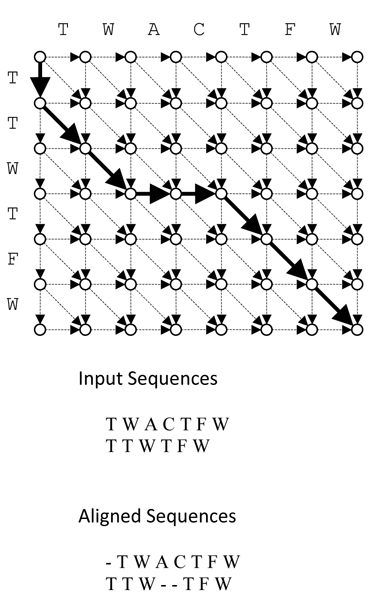
**GAS and RB**.

The Grid Answer Space (GAS), which is the extended version of the grid table used in DP for pair-wise alignment, is a multi-dimensional table with a sequence placed in one of its axes. The use of this table provides a unique one-to-one relationship between any possible answer of a MSA and the associated arrowed line as depicted in Figure 2.

#### Rubber Band

Any answer for a MSA can be presented by one and only one arrowed line. This unique arrowed line for each answer is called a Rubber Band (RB). There are two restrictions on each RB to make a valid alignment, they are as follows:

1. Each RB must start from the upper left corner (0, 0,..., 0) and finishes at the lower right corner (|*S*_1_| + 1, |*S*_2_| + 1,..., |*S*_*N*_| + 1) of the GAS.

2. There cannot be any backward section in each RB. That is, each section can only be diagonal or parallel to the one of its GAS' axes.

#### Primary Pole

A Primary Pole (PP) refers to a fixed point in GAS that the RB is ought to pass by. In fact, PPs are the sections of the GAS that force the optimization procedure to align a predefined number of characters (of each sequence) with each other.

#### Secondary Pole

Secondary Poles refer to grid points in GAS that a RB passes through one-by-one to generate the final answer. Now, PPs can be far apart from one other, however, secondary poles need to be adjacent. This type of poles is only used to connect PPs to each other. For brevity purposes, secondary poles are referred to as 'poles' for the rest of this paper.

#### Primary Pole Score

As described earlier, each PP points out predefined locations of input sequences that need to be aligned with each other. If the related to these locations are augmented in a single string, the Primary Pole Score for that particulate PP is defined as the alignment score of that augmented string (with respect to the scoring matrix used for the whole alignment).

#### Sticky Poles

Sticky Poles (SPs) are imaginary poles in the system related to locations in a GAS with high Primary Pole Scores. That is, the optimization procedure can have a pole with a high Primary Pole Score if it places a PP on that special place. Therefore, each SP is located in a GAS to represent a column from the input sequences to align identical or closely related nucleotides from different input sequences with one another.

#### Alignment Score

In each MSA instant, an Alignment Score is defined to evaluate the quality of each answer. The Sum-of-Pairs Score (SPS) with Penalized Gap Opening is the criterion used in this approach. In SPS, each column in an alignment is scored by summing the scores of all pairs of characters in that column. The score of the final alignment is then summed over all column scores. In the Penalized Gap Opening scheme, there are two factors to calculate the score/cost of a gap: opening and extension. Gap opening is applied to each gap once and the gap extension corresponds to the length of each gap. The cost of a gap opening is usually considered to be 5–10 times more than that of a gap extension [24,25]. The use of two factors in calculating a gap is related to a well-known biological fact that having few longer gaps is more plausible than having several short gaps in an alignment.

### Combination of GA and RBT to solve MSA

**Figure 3:**
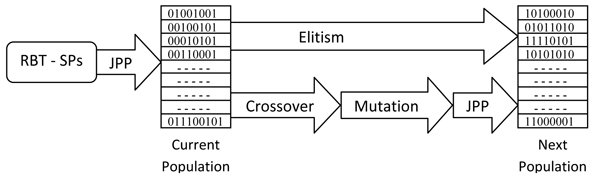
**RBT-GA overall procedure**.

The overall procedure of RBT-GA is depicted in Figure 3. Details are as follows.

#### Chromosomes in RBT-GA

The way the chromosomes are defined represent the most important part of any application of a GA. In RBT-GA, each chromosome consists of a predefined number genes assigned before launching the main algorithm. In RBT-GA, each gene is not a simple binary or decimal number; rather a complex vector representing a PP in the GAS. Therefore, each chromosome is a set of PPs plus two fixed PPs (the first and the last) in the RBT framework. The first and last genes are always fixed to the upper left corner and lower right corner of the GAS, respectively. These two fixed genes guarantee generating a valid chromosome, and therefore a valid alignment.

#### Generate the Initial Population

SPs of the RBT are used to generate the initial population in RBT-GA. The following procedure is designed to generate *N *chromosomes, each with the length of *M*.

Step 1: Locate All Sticky Poles and save in ArrStickyPoles

Step 2: For *N *times, repeat steps 3–8

Step 3:   Prepare a DummyChrm with the length of *M*

Step 4:   For *M *times repeat Steps 5–6

Step 5:      Select a random SP from ArrStickyPoles.

Step 6:      Add SP to DummyChrm

Step 7: Fix DummyChrm

Step 8: Add DummyChrm to Initial Population

In Step 7, every DummyChrm that is made is modified to generate a valid RB. Here, genes of each DummyChrm are reordered so that their PP are guiding the RB from upper left corner of a GAS to its lower right corner without violating any of the two RB's restrictions. If there were several PPs that do not satisfy the mentioned criteria, one or two of them are jammed to each other to fix this problem. This procedure, namely Jam Primary Poles (JPP) as shown in Figure 3, is borrowed from the RBT framework.

#### Genetic Algorithm Operators

Three GA operators are also used for RBT-GA. For Elitism, a predefined percentage of each population is directly copied to the next one. In Crossover, two chromosomes are split from a random point to swap genes between. In Mutation, each coordinate of each gene (PP) of a chromosome is added by a small random number. Note that, after performing Crossover followed by Mutation operators, PPs in each of the generated chromosomes may not satisfy the two major restrictions of a valid RB. Therefore, the Jam Primary Pole procedure is launched again to fix every generated offspring, leading to valid alignments.

#### RBT-GA Termination

RBT-GA's optimization process is terminated when no improvement is met for a predefined number of iterations. The best alignment score for the current population is used as the criterion for this purpose.

#### RBT-GA final tuning

After termination the RBT-GA's optimization process, another procedure is launched for the final tuning of the obtained chromosomes. This process is the Final Tuning procedure which is borrowed from the RBT framework [24,25].

## Results

Normally, when solving MSA problems, the optimal answer is unknown and there is no concrete criterion to evaluate the quality of a given algorithm, unlike the case for Pair-wise alignment where an optimal solution can always be found. Therefore, standard benchmarks, like BALiBASE, are provided to measure the efficiency of MSA algorithms.

The first version of BAliBASE [36] was dedicated to the evaluation of multiple alignment programs and was divided into five hierarchical reference sets of: (1) equidistant sequences with various levels of conservation, (2) families aligned with a highly divergent 'orphan' sequence, (3) subgroups with less than 25% residue identity between groups, (4) sequences with N/C-terminal extensions, and (5) internal insertions. For release 2.0 of BAliBASE, these alignments have been manually verified and corrected by superposition of all known three-dimensional structures, using the lsqman program [37]. In this benchmark, an open source program is also provided to score the quality of each answer by comparing it with the one found manually. The maximum score is 1.0 and is assigned to the alignments that are identical to the benchmark's answer; minimum is 0.0 and is assigned to unrelated/unrealistic answers; and, a number between 0.0 and 1.0 for the others. The score would be higher when the generated answer is closer the manually calculated one.

**Figure 4:**
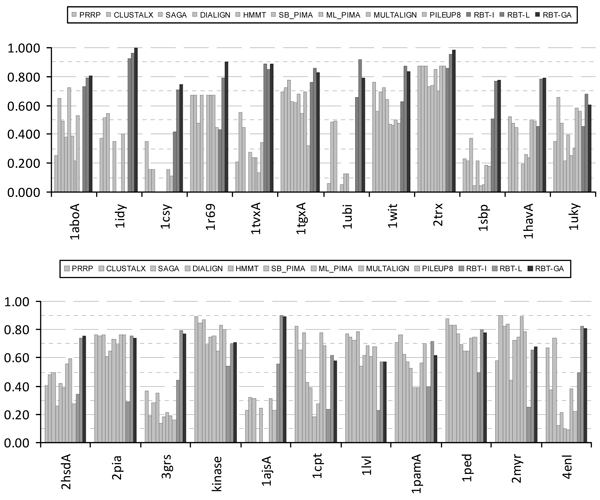
**RBT-GA in Reference #2**.

**Figure 5:**
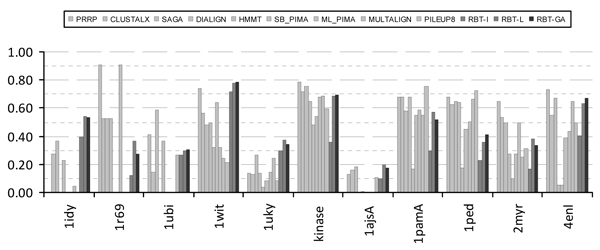
**RBT-GA in Reference #3**.

To demonstrate the performance of the approach proposed in this paper, RBT-GA is used to solve all benchmarks from Reference #2 and #3 of BALiBASE 2.0 [38]. For all these benchmarks, the BLOSUM62 scoring matrix with the gap penalty of 10 and 1 for the Gap Opening and Gap Extension, respectively, is selected. Figure 4 shows the bar graph representation of the performance of RBT-GA compared with other approaches (including our previous approaches RBT-I [24], and RBT-L [25]) formerly designed to solve the stated benchmarks in Reference #2. Figure 5 shows similar results for Reference #3. All RBT-I/L/GA are executed ten times to solve each of these benchmarks. Each algorithm is run ten times to show the robustness of these optimization techniques although they include stochastic optimization steps. For RBT-L, the maximum, average, and minimum of these ten runs are separately depicted to show the robustness of these approaches. For RBT-I, only the best run of these ten executions is reflected; because the other nine answers were not so apart from the best one (always less than %5 in relation to the final Alignment Score); and therefore omitted in these figures. The reason for obtaining different answers for different runs is significantly related to the nature of this optimization process and its operators, as all of them undertake their optimization steps randomly. Therefore, it is not surprising that they will always fall into different parts of the solution space, yet close enough to each other as a sign of the algorithm's robustness and repeatability.

## Discussion

The results obtained by using RBT-GA were quite different and interesting, covering a vast variety of situations. In summary, similar to other approaches formerly presented to solve this problem, although RBT-GA did not manage to find the identical alignments to benchmark answers, it was generally successful. The following sections explain this in more detail.

### Alignment Score vs. BALiBASE score

**Figure 6:**
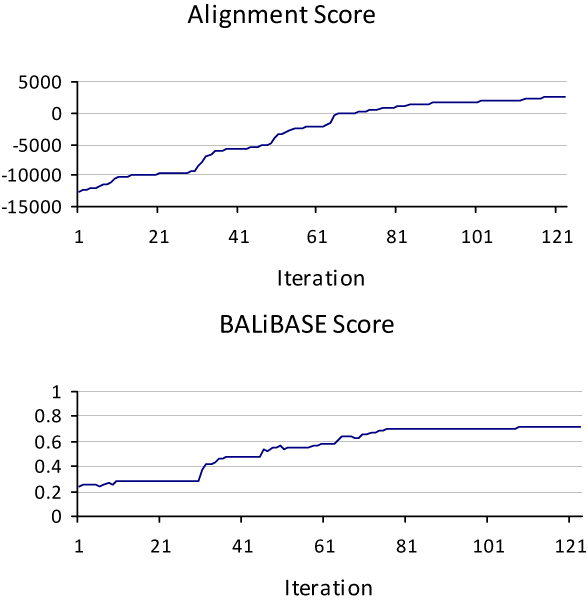
**Alignment vs. BALiBASE Score**.

The first observation made after analyzing the solution trajectory of the benchmarks was the imperfect relationship between Alignment Score, which is purely dependent on the Scoring matrix (BLOSUM62 in this case), and the BALiBASE score, which is purely based on biological facts. However, they seemed to be fairly related. In several cases, gaining higher Alignment Scores yields better BALiBASE scores; although, this cannot be always guaranteed. To investigate this relationship further, we executed the algorithm with different scoring matrices, gap opening and extension values. In almost all cases, the Alignment Score and BALiBASE score showed the same level of uncorrelation. Nevertheless, it seems that in most cases, alignments with higher Alignment Scores have better BALiBASE score as well. Figure 6 shows a sample of this uncorrelation for 1 wit from Reference#3.

### RBT-GA and Reference #2

Reference #2 of the BALiBASE 2.0 is dedicated to 'orphan' sequences. These sequences are significantly different in their number of sequences and their sequences' length. For this class of sequences, RBT-GA has shown different performances. As it was shown in Figure 4, in several cases (like 1idy, 1csy, 1tvxA, 1ubi and 1ajsA), RBT-GA managed to significantly outperform the existing methods. In several other cases, RBT-GA's performance was just fairly comparable to the others, like 1uky, 1tgxA and 2trx. And finally, there were cases that RBT-GA did not significantly outperform the existing alignment methods, like 1cpt, 2myr and 1lvl.

### RBT-GA and Reference #3

Reference #3 of the BALiBASE 2.0 is dedicated to subgroups of sequences with less than 25% residue identity between groups. Performance of RBT-GA in this category was quite different compare to Reference #2. Here, although RBT-GA manages to outperform few of the existing methods, it could not significantly outperform any of them. Overall, the performance of RBT-GA was fairly better than several other algorithms.

### RBT-GA and BALiBASE Score

One of the noticeable facts about RBT-GA answers is its non-zero BALiBASE score at all times. Examining Figures 4 and 5, it can be seen that, several of the existing alignment algorithms have significant performance diversity in their results. For example, Figure 4 shows that PRRP and ML_PIMA received BALiBASE score 0.0 and 0.905 for 1idy and 1r69, respectively. In other words, in these two cases, the final alignment of these two algorithms manifests no biological relationship in one case and almost maximum biological relationship in the other case. In contrast, RBT-GA was always able to identify some biological relationship in the aligned sequences. In some cases, it found the identical answer, such as, 1idy in Reference #2. In other cases where the biological relationship in the input sequences was subtle and no method could find a reasonable answer, RBT-GA performed reasonably, like 1ajsA in Reference #3.

### Overall Performance of RBT-GA

The RBT-GA had a reasonable performance in almost all cases. Although there were instances that some of the existing methods found better solutions, with respect to BALiBASE score, in most cases, the quality of RBT-GA's alignments were as good as or better than the other methods. In some cases, it could even significantly outperform existing methods, such as 1idy and 1ubi in Reference #2.

**Figure 7:**
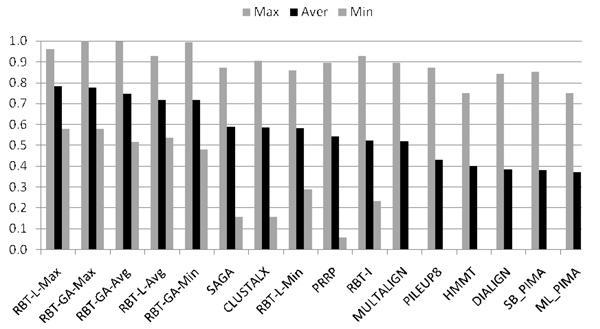
**Overall performance of RBT-GA in Reference #2**.

**Figure 8:**
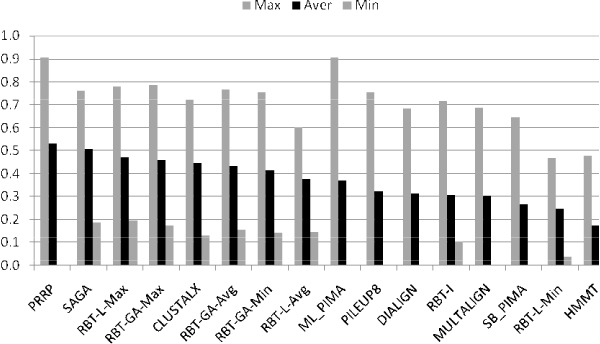
**Overall performance of RBT-GA in Reference #3**.

Figures 7 and 8 show the overall performance of RBT-GA as compared with other approaches. In these figures, the alignment algorithms are sorted according to their average BALiBASE score throughout the whole benchmark. Figure 7 shows that RBT-L-Max slightly outperformed RBT-GA-Max, while RBT-GA-Max significantly outperformed all other methods in Reference #2, almost %20 better than the fourth best, SAGA. Figure 8 shows that RBT-GA-Max is ranked fourth based on its average BALiBASE score in Reference #3, although its average BALiBASE score is just 7% below the best approach, PRRP.

### RBT-GA versus RBT-I/L

Figures 4, 5, 7 and 8 show that RBT-GA significantly outperforms RBT-I. These figures show that even the worse alignment of RBT-GA (RBT-GA-Min) is almost 20% and 10% better than RBT-I for References #2 and #3, respectively.

RBT-L and RBT-GA on the other hand show very similar performances. However, Figures 4, 5, 7 and 8 also show that RBT-GA-Max is slightly worst (around 1%) than RBT-L-Max in both References. In contrast, RBT-GA-Avg is better than RBT-L-Avg for almost 6% in both References. Thus, the worse alignment that each of these techniques has found is used for the sake of comparison. RBT-GA-Min significantly outperforms RBT-L-Min for 14% and 17% in Reference #2 and #3, respectively. Also, in both References, the worse alignment found by RBT-GA is better than the average alignment found by RBT-L.

The above comparisons imply that having so many SPs, biologically meaningful or not, increase the chance of misleading the main optimization algorithm. This could be the reason why RBT-L managed to perform as well as RBT-GA in the best scenarios and significantly performed less efficiently in the worse cases.

These observations imply that the way in which SPs are generated at the start of the main optimization process is the key to better alignments. In conclusion, one can state that the location of an AA is more important than its index in a protein.

## Conclusion

In this paper, a novel approach (RBT-GA) based on the combination of Rubber Band Technique and a Genetic Algorithm is presented to solve the Multiple Sequence Alignment problem. RBT-GA is a population based optimization algorithm that starts from a set of possible answers (initial population), and gradually improves it to find the optimal alignment. In this approach, GA is used to gradually improve the quality of different answers in the population (presented as different chromosomes). Here, genes of each chromosome are in fact RBT's Sticky Poles that are used to identify locations in the input sequences that are most likely biologically related (motifs). RBT-GA is tested by solving several benchmarks from the BALiBASE 2.0. The results showed great promise of the proposed approach.

Based on the promising results obtained from RBT-GA, the future direction is (1) to investigate more optimization techniques to adopt in this framework, and (2) to improve the convergence speed of RBT-GA through parallelization.

## Competing interests

The authors declare that they have no competing interests.

## Authors' contributions

JT and AYZ developed the framework for the new MSA method. JT implemented the framework and developed the associated software and simulations. JT and AYZ wrote and edited different versions of the manuscript.

## References

[B1] Watson JD, Crick FHC (1953). A Structure for Deoxyribose. Nucleic Acid Nature.

[B2] Wang L, Jiang T (1994). On the complexity of the multiple sequence alignment. Journal of Computational Biology.

[B3] Abdesslem L, Soham M, Mohamed B (2006). Multiple sequence alignment by quantum genetic algorithm. 20th International Parallel and Distributed Processing Symposium (IPDPS 2006).

[B4] Needleman SB, Wunsch CD (1970). A general method applicable to the search for similarities in the amino acid sequence of two proteins. Journal of Molecular Biology.

[B5] Barton GJ, Sternberg MJE (1987). A strategy for the rapid multiple alignment of protein sequences. Journal of Molecular Biology.

[B6] Taylor WRA (1988). Flexible method to align large numbers of biological sequences. Journal of Molecular Biology Evolution.

[B7] Devereux J, Haeberli P, Smithies O (1984). A comprehensive set of sequence analysis programs for the VAX. Nucleic Acids Research.

[B8] Thompson JD, Gibson TJ, Plewniak F, Jeanmougin F, Higgins DG (1997). The CLUSTAL_X windows interface: flexible strategies for multiple sequence alignment aided by quality analysis tools. Nucleic Acids Research.

[B9] Thompson JD, Higgins DG, Gibson TJ (1994). CLUSTAL W: improving the sensitivity of progressive multiple sequence alignment through sequence weighting, position-specific gap penalties and weight matrix choice. Nucleic Acids Research.

[B10] Sneath PHA, Sokal RR (1973). Numerical Taxonomy.

[B11] Saitou, Nei (1987). The neighbor-joining method: a new method for reconstructing phylogenetic trees. Journal of Molecular Biology and Evolution.

[B12] Smith RF, Smith TF (1992). Pattern-induced multi-sequence alignment (PIMA) algorithm employing secondary structure-dependent gap penalties for use in comparative protein modelling. Protein Engineering.

[B13] Morgenstein B, Dress A, Werner T (1996). Multiple DNA and protein sequence alignment based on segment-to-segment comparison. Proceedings of the National Academy of Sciences.

[B14] Gotoh O (1996). Significant improvement in accuracy of multiple protein sequence alignments by iterative refinement as assessed by reference to structural alignments. Journal of Molecular Biology.

[B15] Notredame C, Higgins DG (1996). Saga – sequence alignment by genetic algorithm. Nucleic Acids Research.

[B16] Katoh K, Misawa K, Kuma K, Miyata T (2002). MAFFT: a novel method for rapid multiple sequence alignment based on fast Fourier transform. Nucleic Acids Research.

[B17] Do CB, Mahabhashyam MS, Brudno M, Batzoglou S (2005). ProbCons: Probabilistic consistency-based multiple sequence alignment. Genome Research.

[B18] Notredame C, Higgins DG, Heringa J (2000). T-Coffee: A novel method for fast and accurate multiple sequence alignment. Journal of Molecular Biology.

[B19] Edgar RC (2004). MUSCLE: multiple sequence alignment with high accuracy and high throughput. Nucleic Acids Research.

[B20] Pei J, Grishin NV (2006). MUMMALS: multiple sequence alignment improved by using hidden Markov models with local structural information. Nucleic Acids Research.

[B21] Baldi P, Chauvin Y, Hunkapiller T, McClure MA (1994). Hidden Markov models of biological primary sequence information. Proceedings of the National Academy of Sciences.

[B22] Krogh A, Mian I, Haussler D (1994). A hidden Markov model that finds genes in Escheria Coli DNA. Nucleic Acids Research.

[B23] Eddy SR (1995). Multiple Alignment Using Hidden Markov Models. 3rd ISMB.

[B24] Taheri J, Zomaya AY, Zhou BB (2008). RBT-I: A Novel Approach for Solving the Multiple Sequence Alignment Problem. The 6th ACS/IEEE International Conference on Computer Systems and Applications (AICCSA-08); Qatar.

[B25] Taheri J, Zomaya AY, Zhou BB (2008). RBT-L: A Location Based Approach for Solving the Multiple Sequence Alignment Problem. The University of Sydney Technical Reports.

[B26] Zhang C, Wong AKC (1997). Toward efficient multiple molecular sequence alignment: a system of genetic algorithm and dynamic programming. IEEE Transactions on Systems, Man and Cybernetics, Part B.

[B27] Cai L, Juedes D, Liakhovitch E (2000). Evolutionary computation techniques for multiple sequence alignment. Proceedings of the Congress on Evolutionary Computation.

[B28] Thomsen R, Fogel GB, Krink T (2003). Improvement of clustal-derived sequence alignments with evolutionary algorithms. The 2003 Congress on Evolutionary Computation (CEC '03).

[B29] Nguyen HD, Yamamori K, Yoshihara I, Yasunaga M (2003). Improved GA-based method for multiple protein sequence alignment. The 2003 Congress on Evolutionary Computation (CEC '03).

[B30] Hsiao Y-T, Chuang C-L, Chien C-C (2004). A novel GA-based algorithm approach to fast biosequence alignment. IEEE Conference on Cybernetics and Intelligent Systems.

[B31] Liu Lf, Huo Hw, Wang Bs (2004). Aligning multiple sequences by genetic algorithm. International Conference on Communications, Circuits and Systems (ICCCAS 2004).

[B32] Omar MF, Salam RA, Rashid NA, Abdullah R (2004). Multiple sequence alignment using genetic algorithm and simulated annealing. Proceedings of International Conference on Information and Communication Technologies: From Theory to Applications.

[B33] Chen Y, Pan Y, Chen J, Liu W, Chen L (2006). Partitioned optimization algorithms for multiple sequence alignment. 20th International Conference on Advanced Information Networking and Applications (AINA 2006).

[B34] Goldberg DE (1989). Genetic Algorithms in Search, Optimization and Machine Learning.

[B35] Goldberg DE (2002). The Design of Innovation: Lessons from and for Competent Genetic Algorithms.

[B36] Thompson JD, Plewniak F, Poch O (1999). BAliBASE: a benchmark alignment database for the evaluation of multiple alignment programs. Bioinformatics.

[B37] Kleywegt GJ, Jones TA (1995). Where freedom is given, liberties are taken. Structure.

[B38] Nucleic Acids Research [] visited Aug-2007. http://www.pubmedcentral.nih.gov/articlerender.fcgi?artid=29792.

